# Development of a Novel Multi-Channel Thermocouple Array Sensor for In-Situ Monitoring of Ice Accretion

**DOI:** 10.3390/s20082165

**Published:** 2020-04-11

**Authors:** Luke Rieman, Erdogan Guk, Taeseong Kim, Chankyu Son, Jung-Sik Kim

**Affiliations:** 1 School of Mechanical, Electrical and Manufacturing Engineering, Loughborough University, Loughborough LE11 3TU, UK; luke.rieman@gmail.com (L.R.); E.Guk@lboro.ac.uk (E.G.); 2 Bozok Üniversitesi Mühendislik-Mimarlık Fakültesi Erdoğan Akdağ Kampüsü Atatürk Yolu 7. km, 6000 Yozgat, Turkey; 3 Department of Wind Energy, Technical University of Denmark, 2800 Kgs. Lyngby, Denmark; cson@dtu.dk; 4 Department of Aeronautical and Automotive Engineering, Loughborough University, Loughborough LE11 3TU, UK; J.Kim@lboro.ac.uk

**Keywords:** icing, multi-channel thermocouple array (MCTCA), thermocouple

## Abstract

A test was performed to determine the efficacy of a novel multi-channel thermocouple temperature sensor employing “*N+1*” array architecture for the in-situ detection of icing in cold climates. T-type thermoelements were used to fabricate a sensor with six independent temperature sensing points, capable of two-dimensional temperature mapping. The sensor was intended to detect the high latent heat of fusion of water (334 J/g) which is released to the environment during ice formation. The sensor was embedded on a plywood board and an aluminium plate, respectively by an epoxy resin. Three different ice accretion cases were considered. Ice accretion for all cases was achieved on the surface of the resin layer. In order to analyse the temperature variation for all three cases, the first 20 s response for each case was averaged between three cases. A temperature increase of (1.0 ± 0.1) °C and (0.9 ± 0.1) °C was detected by the sensors 20 s after the onset of icing, attributed to the latent heat of fusion of water. The results indicate that the sensor design is well-suited to cold temperature applications and that detection of the latent heat of fusion could provide a rapid and robust means of icing detection.

## 1. Introduction

Aircraft and wind turbines encounter the icing phenomenon when they operate under high humidity and low temperature. Water droplets in the air impinge on the surface of these machines. If convective and/or conductive cooling is sufficient to remove the latent heat of fusion of the water, the water will freeze on the surface. This icing is broadly categorized as rime or glaze ice according to the rate of convective and/or conductive cooling. Rime ice occurs under low-temperature and humidity conditions. The impinged liquid water droplets freeze immediately on contact with a surface with a sufficient cooling rate [1]. On the other hand, glaze ice can be observed under high-temperature and humidity conditions. The impinged water droplets form a thin water film on the surface, which then freezes more slowly. However, most icing conditions are situated in mixture ice that cannot be clearly distinguished between rime and glaze ice [2].

In-flight icing usually occurs when the aircraft passes through clouds containing supercooled liquid water droplets, which can freeze on surfaces such as the wings, fuselage, and engine inlet. Ice accretion can change the shape of the leading edge of the aerofoil, resulting in significant degradation of the aerodynamic performance. When aircraft are exposed to severe icing conditions for a long time, ice threatens the controllability and flight safety, as it reduces the stall margin and increases the stall speed and causes an abrupt drop in pitching moment. The icing on the engine intake deteriorates the performance of the engine due to the distorted flow of intake air. If ice projectiles, which are shed from the surface, are sucked into the engine, it can cause engine failure. Forty aviation accidents from the period 2006 to 2010 were directly related to in-flight icing occurring on the wings, fuselage, or control surfaces [3]

The installation of wind turbines in cold climates or at high elevations is often desirable due to the availability of land, low population density, increased power due to higher air density, and greater wind speeds with increasing elevation [4,5]. Such sites are frequently subject to atmospheric icing conditions which may lead to accretion of ice on the surface of turbine rotor blades, [1,6,7,8,9]. As with aircraft, aerofoil icing significantly impacts the aerodynamics of the blade, leading to power performance losses of up to 40% [9,10]. Ice build-up also leads to increased fatigue loading and may significantly reduce gearbox lifetime due to mass imbalance [11]. Furthermore, blade icing may pose a significant safety risk as large ice chunks can be thrown up to 1.5 times the turbine tip height, endangering workers and the public [12]. Turbines must typically be shut down during icing events to prevent fatigue loading or to facilitate access for workers, further compounding energy losses [4,13].

To mitigate the effects of icing and to ensure aviation safety, ice protection systems have been equipped to aircraft and wind turbines. Ice protection systems require ice detection systems and anti-/de-icing systems with logical algorithms. Several methods for anti-/de-icing systems have been suggested: fluid application, thermal melting, mechanical removal, and hydro-/ice-phobic coatings. The fluid application method prevents icing by applying a chemical solution such as ethylene glycol. The chemical solution lowers the freezing point of the impinged water [14]. With thermal melting methods, electro-thermal pads are inserted between the surface layers, or hot air is circulated inside the wing or blade [15]. To physically remove the accumulated ice on the surface, various devices can be used, such as electro-vibratory, microwave, shape memory alloy, pneumatic boots, and electro-impulsive [16]. Hydro-/ice-phobic coatings reduce the rate of ice accretion and aid in ice shedding [13]. Considering the technical maturity, the weight of systems, the environment, durability, and actual effects, thermal melting methods are mainly used as the anti-/de-icing systems for aviation [15] and wind energy [17] engineering. 

Anti-/de-icing systems are controlled based on measured data from the ice detection systems to determine the operating time, intervals, and intensity. A reliable ice detection system is therefore essential, and two or more ice detection systems have been applied in aviation and wind energy fields. In the aviation field, ice detectors can be classified into two categories: icing condition detectors and ice accretion detectors. Icing condition detectors monitor the ambient liquid water contents (LWC) and temperature [18,19]. Dual wet and dry heated elements, hot wire, radiometer [20], and optical backscatter [21] were developed for this purpose [22]. Rather than measuring the actual ice accretion, these indicate the potential for ice accretion. On the contrary, the following ice accretion detectors measure the build-up ice on the specific surface: vibration [23,24], electro-thermal [22], electro-optical [25], fiber-optical [26], radio frequency [27], micro-mechanical sensor [28], inductive devices [29], and ultrasonic [30]. An arc lamp is used to illuminate the aircraft wings to enhance regular visual inspection [31]. 

Currently adopted ice detection systems in the wind energy industry are also icing condition detectors and ice accretion detectors [32]. Wind turbines can take advantage of ice accretion detectors due to the large interior space of the blades and fewer weight constraints. There have been various attempts for ice detection systems of wind turbines including those methods used in the aviation field. As well as icing condition detectors, power-curve [33], dew point [34], heated and unheated anemometers [35], and noise frequency [36] are monitored. There have been various attempts to produce ice accretion detectors for wind turbines, such as impedance sensing [37], capacitance [38], or resonant frequency [39] on the blade. Infrared [40] or web cameras [36] are also applied to visually identify icing on the blade.

Although a lot of devices were developed and demonstrated as listed above, important issues were reported. Richards, T. [41] reported that aircraft icing sensors use an indirect means of ice detection which is nearly always mounted on the fuselage and distanced from the ice accretion surface of importance, such as the wings and engine intake. Homola, M. et al. [32] also emphasized that the best position for the ice detection system of wind turbines is on the blade itself, and as close to the tip as possible. However, attaching ice detection sensors to the exterior surfaces of wings and engine intakes of aircraft or to the blade tips of wind turbines can disrupt the flow field. Consequently, the overall performance of aircraft and wind turbines will be degraded in normal conditions.

For these reasons, military aircraft mainly adopt the electro-thermal ice detector [22]. Thin wires with resistance are attached to the specific surface in the array of a protective groove. When icing is expected, power is applied to the wire and the sensor records the wire temperature. If a constant temperature range is observed due to the heat of fusion of ice, even when constant power is steadily applied, an ice detection signal is provided. However, they still suffer from the following problems. Firstly, it is difficult to apply the electro-thermal detector to a wide area. Although the electro-thermal ice detector can be attached to any surface, the grooves to protect the wires increase the surface roughness which in turn affects the flow near the surface. Secondly, it takes a certain amount of time to detect icing again because the wire and the surface temperature increase above the freezing temperature during the detection. Finally, it is dependent on other ice detection systems as an active device, although this sensor can accurately detect the icing at a certain area.

Infrared cameras [40] are used to detect the ice accretion on the wind turbine blades. By measuring the surface temperature, the latent heat of water can be detected. However, the infrared cameras attached to the blade surface also cause disturbances in the flow field. Furthermore, it is very expensive and extra protection systems for humidity, dust, bugs, etc., should be provided. It only measures the temperature of the outer ice layer, not the exact surface temperature. The atmospheric transmission such as distance, atmospheric temperature, and relative humidity should be correlated. Since the ambient temperature and relative humidity can be obtained by the icing condition detectors, infrared cameras are also dependent on the icing condition detectors. 

To this end, this study suggests a novel ice detection sensor by using a proprietary multi-channel thermocouple array (MCTCA) technology [42,43,44,45,46,47,48,49,50] by measuring the latent heat of fusion, which is the unique physical characteristic when liquid water turns into ice. Thermoelements can be arranged in an array or grid configuration with each node spot-welded or soldered to produce a thermocouple (TC) junction. This has the advantage of reducing the number of thermoelements required to “*N+1*”, where “*N*” is the desired number of TC junctions or “sensor sensing points” (SSPs) in the array. In comparison, conventional TC architecture requires “*2N*” thermoelements for “*N*” TC junctions. Therefore, the benefit of the “*N+1*” architecture from space and material savings scales significantly with the number of SSPs. Thermocouple technology is robust and well-understood, and “*N+1*” MCTCA architecture represents a versatile, low-cost method for fast, accurate temperature mapping over a wide range of operating temperatures [42]. 

The accuracy of the “*N+1*” architecture is governed by the same variables as conventional “*2N*” architecture. Most notably, the Seebeck coefficient of each thermoelement should be uniform throughout its length. The introduction of additional junctions in the “*N+1*” architecture necessarily creates “dissimilar regions” where the Seebeck coefficient is altered across the width of the junction by the presence of the second thermoelement material. It has been shown that where the junction size is small relative to the junction spacing, the accuracy of the “*N+1*” architecture is very close to that of conventional TCs [42].

An ice detection sensor using the MCTCA can expect the following advantages. First, the MCTCA detects ice events with relative temperature changes between structure surface temperature and the latent heat of fusion due to the icing events. Therefore, it is not affected by different temperature distributions along the considered structures and/or ambient temperature conditions due to convection, rotation, etc. Second, the thin and flexible MCTCA is simple in structure and easily implemented on a curved surface such as the leading edge of turbine blades, aircraft wings, and engine intakes without any disturbance of the flow field. To create a smooth surface, the thin wire array can be covered by resin or insulted between the surface layers, unlike the electro-thermal sensors which require protection grooves and infrared cameras attached on the outer surfaces. Third, the MCTCA is able to detect the presence or absence of icing as well as the distribution of ice over a wide area of the wing/blade. Therefore, an ideal power distribution can be suggested for the anti-/de-icing systems. Fourth, the rate of false-positives is intrinsically lower with this detection method because it can only detect the formation of ice; liquid water, dust, insects, etc., produce no signal. Finally, the MCTCA can be designed as an independent active ice detector. The MCTCA can accurately measure the surface temperature to detect the latent heat of fusion [51] like the electro-thermal ice detector and infrared cameras. Since a thermocouple array is located just below the surface layer, unlike infrared cameras that require the meteorological parameters, the MCTCA can accurately predict the surface temperature without other external sensors. The low-power MCTCA can measure the surface temperature in real time with high sensitivity and adequate response. The MCTCA does not require the resting time to heat the surface above the melting point and recover back to ambient temperature, while the electro-thermal sensors need assistance from other sensors during the resting time.

This study was performed to verify the feasibility of the MCTCA as an ice detection sensor. The accuracy and response time of the sensor was investigated at low temperatures such as rime ice conditions in a climate chamber. To consider the attachment on various surfaces, the dependency of the surface material was analysed. The performance of the sensor when embedded in resin to create a smooth surface was also evaluated.

## 2. Methodology

### 2.1. Multi-Channel Thermocouple Array (MCTCA) Architecture

**Figure 1:**
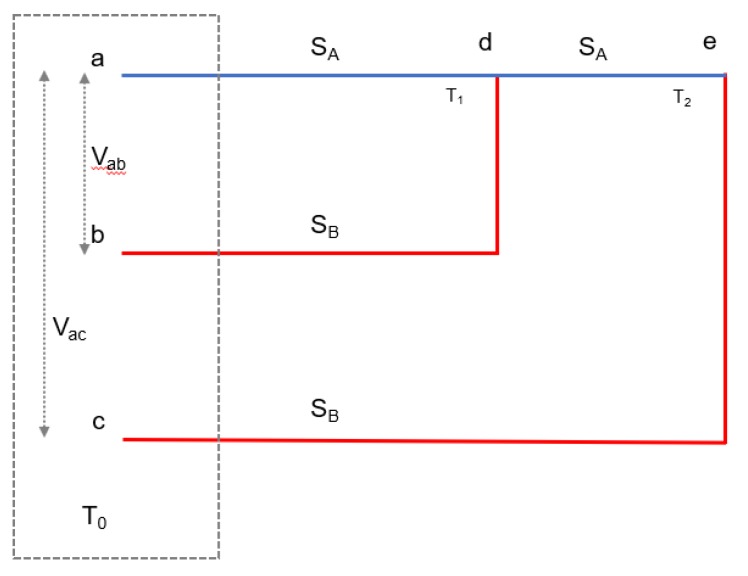
Schematic diagram of recursive unit for “*N+1*” array architecture. The red and blue lines indicate two different thermoelement types. Each red line may be a different material, however in this study only two distinct thermoelement materials were used. Here ‘*V_ab_*’ and ‘*V_ac_*’ are the resultant thermocouple (TC) voltages produced by a temperature gradient between the ‘cold junctions’ (*a*,*b*,*c*) which are maintained at ‘*T*_0_' , and the ‘hot junctions’ (*d*,*e*) with temperatures ‘*T*_1_' and ‘*T*_2_' respectively.

MCTCAs obey Seebeck theory, as do conventional TCs, and their output voltages can be reconciled to infer the temperature at each SSP. Figure 1 shows a schematic diagram of an MCTCA. For a single thermoelement, the Seebeck potential ‘*V*_emf_’ created between its ends when a temperature difference exists between them is given by:

(1) $$V_{emf}=\int_{T_{cold}}^{T_{hot}}S\left(T\right)dT$$

where ‘*T_hot_*’ and ‘*T_cold_*’ are the temperatures at either end of the thermoelement and ‘*S(T)*’ is the Seebeck coefficient, defined as:

(2) $$S\left(T\right)=\frac{dV}{dT}\approx \frac{\Delta V}{\Delta T}.$$

When two different thermoelements are combined to form a TC, the temperature-dependent voltage as measured at the ‘cold junction’ of the TC is given by:

(3) $$V_{emf}=\int_{T_{0}}^{T_{1}}S_{A}\left(T\right)dT-\int_{T_{1}}^{T_{0}}S_{B}\left(T\right)dT=\int_{T_{0}}^{T_{1}}\left(S_{A}\left(T\right)-S_{B}\left(T\right)\right)dT$$

where ‘$V_{emf}$’ is the Seebeck potential, ‘$S_{A}$’ and ‘$S_{B}$’ are the Seebeck coefficients of the two thermoelements, ‘*T*_0_' is the ‘cold junction’ temperature at the data logger and ‘*T*_1_′ is the ‘hot junction’ temperature at the sensing point.

When the Seebeck coefficient of a material is known over its operational temperature range, the integral in Equation (1) can be approximated as:

(4) $$V_{emf}=S\left(T_{hot}-T_{cold}\right).$$

Equation (3) then simplifies to:

(5) $$V_{emf}=S_{A}\left(T_{0}-T_{1}\right)-S_{B}\left(T_{0}-T_{1}\right)=\left(S_{A}-S_{B}\right)\left(T_{0}-T_{1}\right)$$

therefore, the voltage ‘*V_ab_*’ measured between ‘*a*’ and ‘*b*’ due to a temperature gradient with ‘*d*’ is given by Equation (6):

(6) $$V_{ab}=V_{ad}-V_{bd}=S_{A}\left(T_{0}-T_{1}\right)-S_{B}\left(T_{0}-T_{1}\right)\;=\left(S_{A}-S_{B}\right)\left(T_{0}-T_{1}\right)$$

where *‘V_ad_’* and *‘V_bd_’* are the thermoelement’s individual electromotive forces (EMF) between ‘*a*’-‘*d*’ and ‘*b*’-‘*d*’ respectively (see Figure 1). Similarly, for an additional TC junction ‘*e*’ at temperature ‘*T*_2_’:

(7) $$V_{ac}=V_{ad}+V_{de}-V_{ce}=S_{A}\left(T_{0}-T_{1}\right)+S_{A}\left(T_{1}-T_{2}\right)-S_{B}\left(T_{0}-T_{2}\right)\;=\left(S_{A}-S_{B}\right)\left(T_{0}-T_{2}\right).$$

Equations (6) and (7) demonstrate how each junction of the TC array behaves as if it were a separate TC. A detailed explanation about the characteristics/working principles of the *‘N+1*′ configured sensor was studied in the authors’ previously published works [48].

### 2.2. Material Selection and Sensor Fabrication

ISO standard T-type copper (0.51 mm) and constantan (0.51 mm) (Cu:Ni/55:45 by wt.) thermoelements were chosen, as these offer high accuracy in the −40 to 125 °C temperature range over other commercial types, e.g., a sensitivity of ± 0.5 °C compared to ± 1.1 °C for K-type TCs [52]. All extension wires and connectors comprised the same thermoelement materials as used to form the sensor junctions, to avoid possible parasitic voltage effects from the creation of additional TC junctions between the sensor and the data logger.

**Figure 2:**
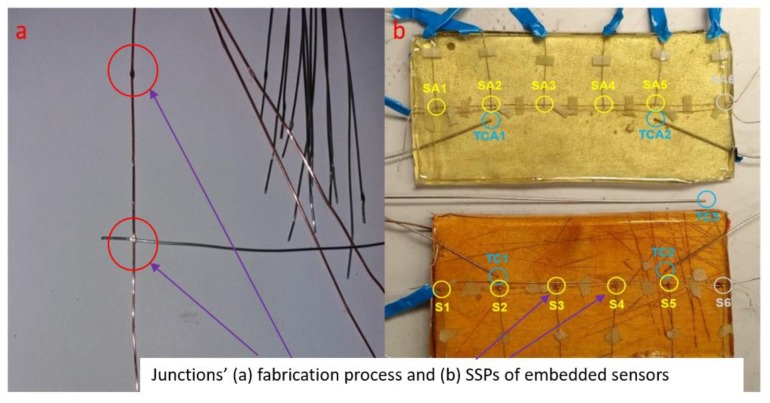
(**a**) Wire just before soldering process, (**b**) aluminium (top) and wood (bottom): mounted sensors following embedding in epoxy resin, showing the location of sensor sensing points and thermocouples. The wire seen running parallel to the copper line is a constantan wire which was not electrically connected to the multi-channel thermocouple array (MCTCA) during the experiment. SA6 and S6 developed a fault and were excluded from the results.

The sensor architecture employed in this study comprised a single copper ‘common line’ thermoelement, to which 6 constantan thermoelements were soldered with a typical separation of 35 mm (see Figure 2a,b). While previous high-temperature applications [47,48] used spot-welded TC junctions, the decision was made to fabricate the sensors in this study using soldered TC junctions due to their improved mechanical stability and because the sensor would not be exposed to high temperatures which would compromise the soldered junction. The large separation of the junctions (35 mm) relative to their size (<1 mm) (Figure 2a) ensures that the temperature gradient across any junction should be tiny, minimising parasitic voltage effects. Alteration of the Seebeck coefficient at the thermoelement junctions and the size of the “dissimilar region” over which this alteration occurs can be considered as the two critical parameters for fabrication-based errors. The error due to sensor fabrication and its array architecture as a result of potential impact of array junctions (which share a common line (array form)) on each other was numerically calculated as ± 0.07 °C via a MATLAB simulation of over 50,000 iterations. 

To assess the impact of the underlying material on the sensor response, particularly in relation to thermal inertia and conductivity, a MCTCA of similar architecture was mounted atop each of a 195 × 100 × 10 mm plywood board and a 212 × 102 × 3 mm aluminium plate. Each SSP was labelled S1-S6 (wood sensor) and SA1-SA6 (aluminium sensor), as shown in Figure 2b. Commercial T-type TC’s labelled TC1/TCA1 and TC2/TCA2 were placed next to S2/SA2 and S5/SA5 respectively for validation purposes. Each sensor together with the commercial TCs was embedded in epoxy potting resin (~2 mm thickness) to provide mechanical stability and to ensure direct contact was maintained between the sensor and the underlying material. An additional commercial TC (labelled TC3 in Figure 2b) was placed alongside the two sensors in the chamber and was used to accurately measure the internal temperature and to determine when the temperature of the sensors had equalised with their surroundings.

**Figure 3:**
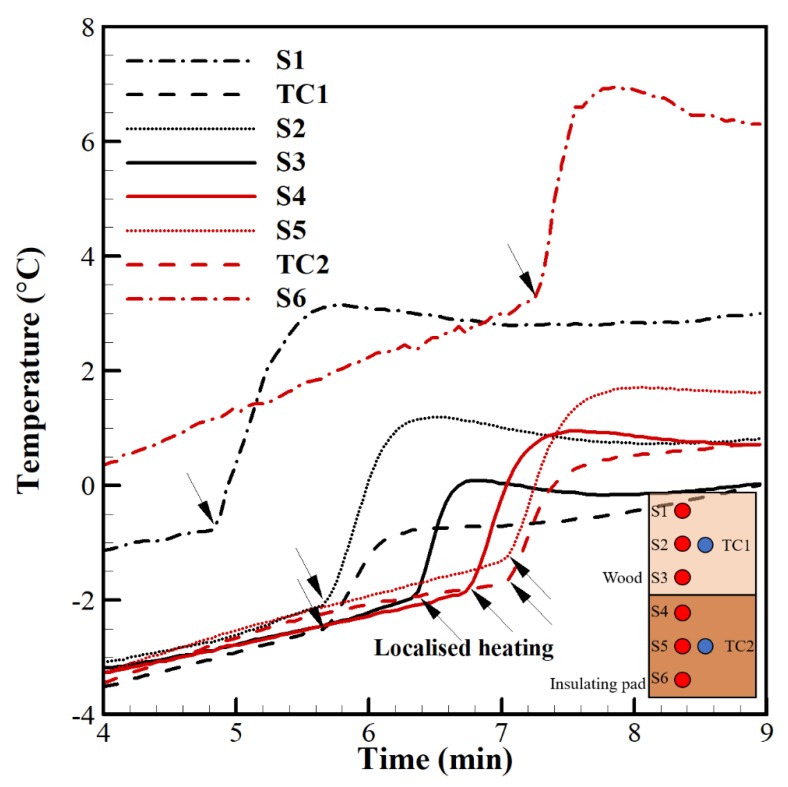
Bench testing of multi-junction thermocouple array demonstrating independence of SSPs temperature measurements. Arrows mark the application of localised heating.

An operator’s finger was used as a heat source to apply localised heating to each of the SSP’s in order to test their individual responses. Figure 3 reveals that all measured temperatures from all SSPs are independent. TC1 responds in accordance with S2, and TC2 responds in accordance with S5 because these are situated in close proximity. The temperature increase varies for each SSP due to the variable time interval of each finger touch. S1 is initially at a higher temperature due to being touched prior to the time shown. S6 displays behaviour indicating the presence of a weak junction, including abnormally large temperature fluctuations and a large temperature offset. It was determined that both S6 and SA6 were damaged during fabrication and their outputs were excluded from the experimental analysis. The damage can most likely be attributed to excessive flexion of the resin layer during curing and should be mitigated by improvements made to the embedding process since this study.

Detailed numerical calibration via mathematical simulation and experimental calibration using calibrated thermocouples (which are certificated by United Kingdom Accreditation Service) were carried out to analyse the potential error across the MCTCA. As a result of these calibration studies, the size of the “dissimilar region” and changes in Seebeck coefficient of the material were found as the two most critical parameters that can cause error. More details about calibration methods applied in this paper are described in Ref. [42]. The experimental calibration test (see Figure 3) was carried out to demonstrate independent readings of the MCTCA SSP’s and to monitor if there is any relatively abnormal reading due damage occurring on the fabricated sensor during implementation. The results show that both the Seebeck coefficient and the size of the “dissimilar regions” were well-controlled during our fabrication. This is because the junction size was minimised as much as it could be which led to a negligible amount of fabrication sourced error. As a result, the total measurement error is still less than 1.1 °C which is comparable with many sensors error (ISO).

### 2.3. Measurement Set-Up

MCTCA and commercial TCs measurements were recorded with a mean frequency of 0.320 Hz using an NI 9213 data logger and a proprietary LabVIEW based data logging software developed by J.-S. Kim et al. [42,43,44,45,46,47,48,49,50].

The measurement error of T-type thermocouples with the NI 9213 data logger is less than ± 1 °C with the 0.02 °C measurement sensitivity at the temperature range of −40 to 70 °C [53]. The LabVIEW software facilitates real-time graphing of SSPs and TC measurements for almost all thermoelement types including K-type and T-type, as well as simultaneous logging of all measurements to an output file with system timestamps at 1 ms resolution. The software is theoretically capable of recording at up to 1 Hz, but in practice is limited by computer hardware. During testing, the achieved temporal resolution was 3.1 s. The error in the data recording interval is negligibly affected by the speed of the PC, as the standard deviation between the timestamps is found as 0.0056 out of 30 samples whilst the corresponding standard error is 0.001. 

**Figure 4:**
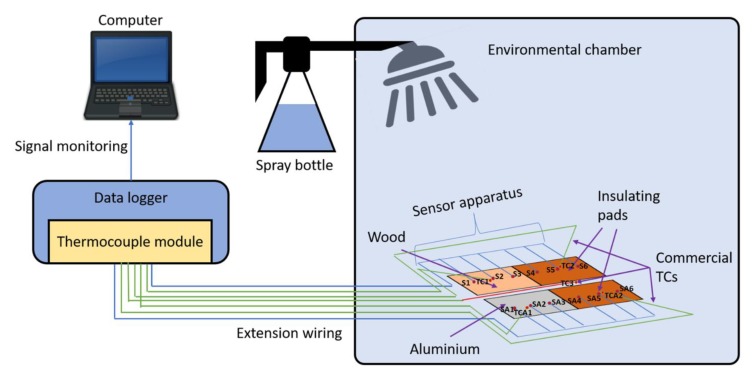
Experimental setup showing the position of the two sensors, each with 50% surface insulation. The upper port was used to extend the spray gun nozzle into the chamber interior during testing. The lower port was used to route the extension cables to the data logger (off-camera), which was directly connected to a laptop PC via USB.

Testing was performed within a Sanyo Gallenkamp HCC065.PF4.MOD environmental chamber. A sketch of the experimental set-up is illustrated in Figure 4. For the duration of each testing set, the chamber temperature setpoint was maintained at −15 °C. This temperature was chosen to ensure sufficient heat loss of the water droplets to the internal environment such that they would be supercooled prior to settling on the sensor surface and icing occurred near-instantaneously. 

An insulating pad consisting of ~4 mm thickness cardboard wrapped in thin polythene sheet was applied to one half of each sensor apparatus, such that three thermocouple junctions (S1/SA1, S2/SA2, S3/SA3) were exposed to direct ice accretion on the surface of the resin, while three were insulated from direct contact with the water droplets (S4/SA4, S5/SA5, S6/SA6). The two sensors were placed in a mirror image configuration as shown in Figure 2 so that they could be placed adjacent to each other without interference from the external connection wires.

**Figure 5:**
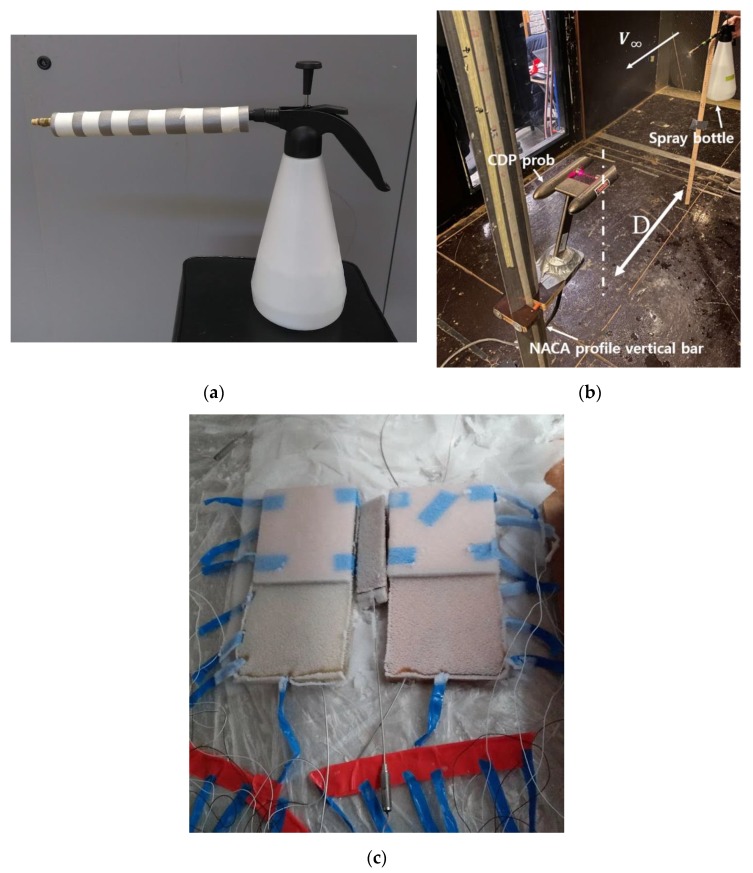
(**a**) Modified spray gun showing nozzle extension and insulation, (**b**) water droplet characteristic measurement set-up, (**c**) Ice formation on sensors showing rime ice accretion.

A fine water mist was generated using a spray bottle modified with an extended nozzle to allow injection through the upper port of the environmental chamber (Figure 5a). The water droplet characteristics generated by the spray bottle such as Median Volume Diameter (MVD) and Liquid Water Contents (LWC) were measured with a Cloud Droplet Probe (CDP) [54]. The CDP measures the diameter of each droplet and counts the number of droplets in 1μm increment from 2 to 50 μm. The CDP calculates the LWC by dividing the total mass of water by volume. The determined volume is depending on the freestream velocity. The averaged flow speed inside the environmental chamber is approximately 1–2 m/s. Therefore, two different freestream velocity conditions, 2 m/s and 4 m/s, are considered to measure the LWC. Figure 5b illustrates the water droplet characteristic measurement set-up. The CDP is mounted on a NACA profile bar vertically fastened. The spray bottle is placed 0.35 m away from the CDP (D = 0.35 m), The LWC, MVD, and distributions of the droplet cloud are measured for 10 s and recorded every 1 s. The measurement of each condition (case 1: $V_{\infty }=2\;m/s$ and D=0.35 m, case 2: $V_{\infty }=4\;m/s$ and D=0.35 m) is repeated for 5 times and the measured LWC and MVD are averaged to obtain the final LWC and MVD. The water droplet characteristics generated by the spray system are shown in Table 1.

Before the water was sprayed, ice cubes were placed inside the bottle to maintain the water temperature close to zero, and insulation was applied to the copper nozzle extension to minimise heat losses to the environment. A 10 s spray was discarded immediately prior to each test to clear the internal pipes and ensure that all water had come directly from the iced reservoir. Commercial TC measurements confirmed that the water exit temperature was (1.0 ± 0.5) °C. During each test, the spray nozzle was inserted a fixed distance into the chamber and held straight. Spraying in this way produced a wide (>0.5 m) coverage of consistent water droplet characteristics at the bottom of the chamber. The sensors were placed at the centre of this area to ensure the most even distribution of icing across the sensor areas (Figure 5c). Three test cases were conducted and their distinct regions for SPPs are shown in (a,b) of Table 2.

Spraying was repeated for durations of up to 60 s with rest periods in between to allow heat to dissipate. Repeated spray events were chosen in favour of continuous spraying because the cooling power of the chamber was insufficient to support sustained ice formation with the considered water droplet characteristics. 

After closing the chamber, the temperature was set to −15 °C and the apparatus was allowed to equalise. SSP and TC measurements were monitored to ensure readings had settled to a steady state before commencing the spray injection. Following the first spray event, the temperature was allowed to return to an approximately steady state in order to fully characterise the sensor response (defined as a temperature change of < 0.01 °C/ min). For all subsequent spray events, the sensor was only partially allowed to return towards equilibrium. This was undertaken to reduce the time required between tests and because we expected the heat input from the latent heat of fusion to be independent of absolute temperature. The total ice mass accreted on each sensor during the full test duration ranged between 27 g and 54 g, dependent on which test case.

The specific heat capacity of water, ‘*c_w_*’, is 4.187J/g*∙*K and the specific heat of ice, ‘*c_i_*’, is 2.108 J/g∙K. The maximum sensible heat input to the system due to the injection of water at (1.0 ± 0.5) °C when the chamber is −15 °C is given by

(8) $$\frac{Q}{m}=c_{w}\Delta T_{1}+c_{i}\Delta T_{2}=35.8\;J/g$$

where ‘*Q/m*’ is the sensible heat per unit mass, ‘*ΔT*_1′_ is the temperature change from 1 to 0 °C, and ‘Δ*T*_2′_ is the temperature change from 0 to −15 °C. This figure does not account for heat lost to the chamber interior. Comparatively, the latent heat of fusion for water is 334 J/g [55]. Thus, where ice formation occurs under these test conditions, the temperature increase at the sensor surface should be largely due to the latent heat of fusion, whilst only a minor contribution from sensible heat could be foreseeable.

## 3. Results and Discussion 

### 3.1. Performance of Proprietary MCTCA Sensor Compared to Commercial Thermocouples

**Figure 6:**
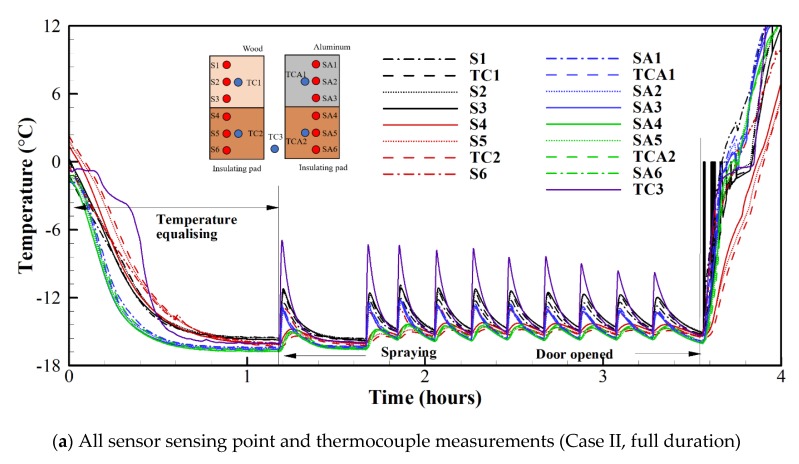

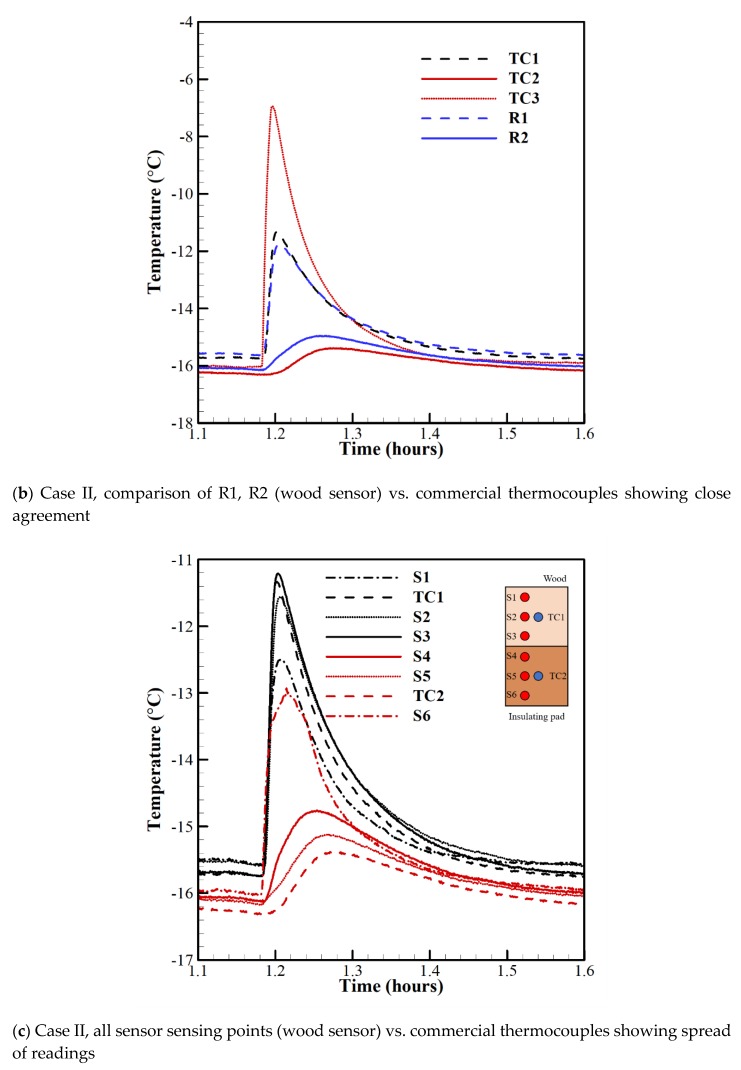
Real-time measured surface temperature.

The outputs of the two sensors were each divided into two “regions” as described in (b) of Table 1, corresponding to the placement of insulation at the surface. Figure 6a shows the output of all SSPs and commercial TCs for the full duration of Case II (see (a) of Table 1). During the first 70 min of the recording, the chamber is cooling to its set point temperature (−15 °C) and the apparatus temperature is equalising. Although this study measured the surface temperature in real time for four hours as shown in Figure 6a, the low-power MCTCA can be operated for a much longer time. The surface temperature can be measured continuously by the MCTCA for a long time without the aid of other equipment. It can be inferred that the MCTCA can be used as an independent active icing detection system since it operates continuously and does not need to be triggered by a secondary sensor as in the case of, e.g., electro-thermal ice detection.

After spraying the water, it is clearly seen that the temperatures are increased rapidly. Based on the predicted heat inputs from both the sensible and latent heat, we believe that this increase is mainly due to ice accretion. Figure 6b shows the response of R1 and R2 compared with the commercial TC measurements during the first spray event of Case II. There is excellent agreement between R1 and TC1 (< 0.7 ± 0.1) °C and between R2 and TC2 (<0.7 ± 0.1) °C. Notably, R2 responds more rapidly and with greater sensitivity than TC2. This might be due to the reduced thermal mass of the SSPs compared to the much larger commercial TCs. Moreover, it is clearly seen that temperature of R1 is increased almost 4 °C due to the latent heat of fusion resulting from the ice accretion. TC3 responds slightly faster and with approximately double the temperature change seen by R1 and TC1, about 9 °C. This indicates that embedding a TC/MCTCA within the resin reduces the temperature increase detectable from icing by approximately 50%.

The composite material using resin is widely applied for aircraft and wind turbines. This study confirmed that the MCTCA can be attached to such a composite surface or inserted between composite layers of the surface and thus not induce any disturbance of the flow fields. Although the insulating properties of the resin negatively impact sensitivity and response time, the MCTCA could still accurately predict the increase in the surface temperature due to latent heat. If the thermal conductivity and the thickness of resin can be correlated, the MCTCA can predict surface temperature more accurately. This will be further investigated in future work.

As shown in Figure 6a, there exists a range of temperature readings of (1.2 ± 0.1) °C prior to the first spray event, which may be attributed to (i) the apparatus not having reached a steady state, (ii) the existence of a thermal gradient within the chamber due to variation of air currents at different points, and (iii) errors in the SSP and TC measurements due to modification of the Seebeck coefficients at connection points or TC junctions. Since the level of agreement is close to the expected T-type TC accuracy at this temperature (±0.5 °C), and since the ice detection capabilities of the sensor rely on relative temperature change rather than absolute temperature, this level of agreement is accepted. Nevertheless, greater consistency during the fabrication of the SSPs may improve this level of agreement, particularly with the soldering process.

Figure 6c shows the individual SSPs constituting R1 and R2 alongside commercial TC readings. Immediately prior to the first icing event, S1, S2 and S3 are in close agreement (<0.2 ± 0.1) °C and S4 and S5 are in close agreement (<0.1 ± 0.1) °C. At their peak temperature change, S1, S2 and S3 deviate by (<1.4 ± 0.1) °C and S4 and S5 deviate by (<0.5 ± 0.1) °C. This can be inferred to (i) uneven resin depth/material surrounding the SSPs and (ii) uneven ice accretion across the sensor area (although this effect is expected to be small). This experimental observation shown in Figure 6b,c shows that the MCTCA can capture the temperature rise within 5 s due to its close proximity to icing surface which reduces the impact of surface materials which have different thermal conductivities. 

### 3.2. Characterisation of Ice Detection Response

**Figure 7:**
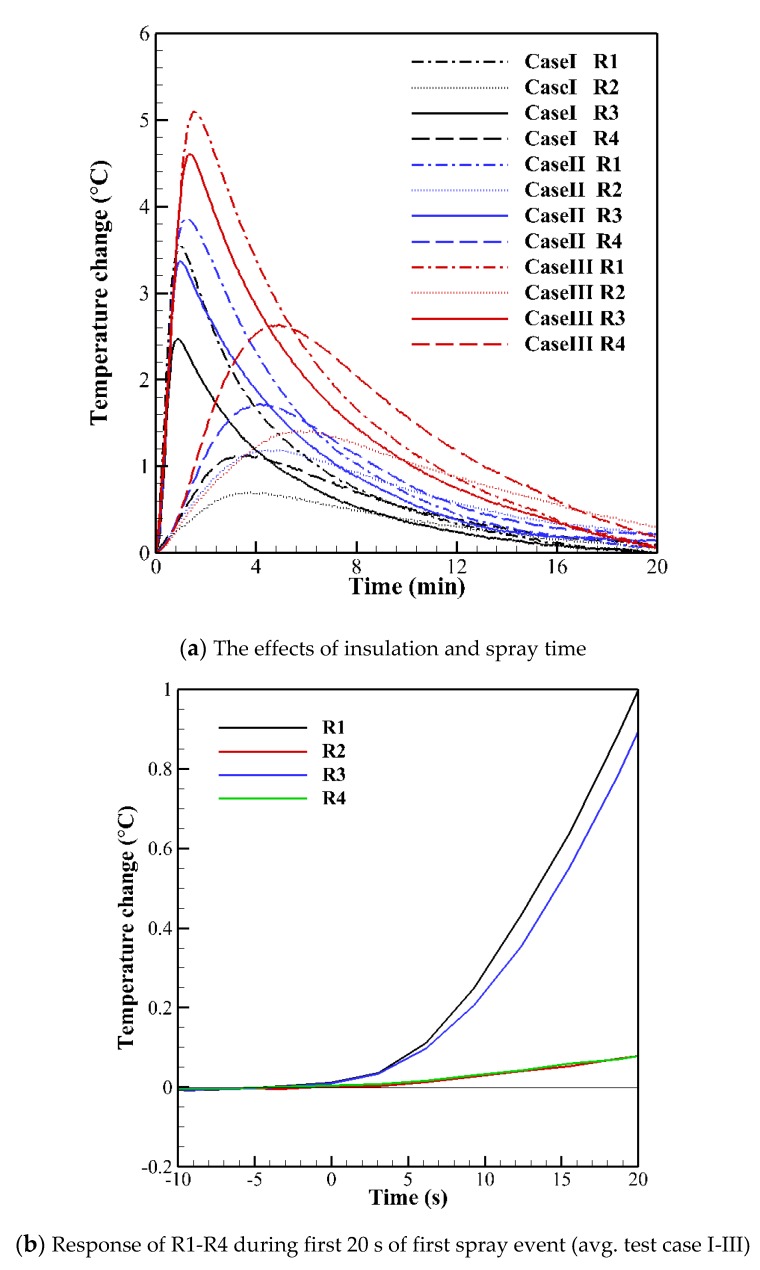
Response of R1-R4 to the first spray event of each test case.

The full response of the first spray event for each test case is shown in Figure 7a. Unsurprisingly, the temperature change increases when the spray time is longer. This is due to the larger water mass deposited at the sensor surface which leads to a greater heat input from the latent heat of fusion during ice formation. More notably, R1 sees a larger temperature change than R3 for all test cases, while R2 sees a smaller temperature change than R4 for all test cases. This may indicate that there is a greater degree of lateral conductive heat transfer from R3 to R4 than from R1 to R2, i.e., greater through the aluminium plate than the plywood. This would have the effect of lowering the temperature rise at R3 and increasing it at R4. 

Since the rate of water deposition to the sensor surface(s) from the spray mist is considered to be constant, the first 20 s of spraying (equal to the full duration of Case I) of the first spray event (where no pre-existing ice is present) should be comparable in each test case. Therefore, the response of each region may be averaged between Case I, Case II and Case III as shown in Figure 7b. The effects of the insulating pads can clearly be seen, with a temperature change of only (0.08 ± 0.03) °C detected by both R2 and R4 in the first 20 s, while R1 and R3 detect an increase of (1.0 ± 0.1) °C and (0.9 ± 0.1) °C respectively over the same period. We attribute this reduction to a modification of the thermal gradient between the sensor and the icing site due to the applied insulation. This suggests that excessive slow build-up of accreted ice may inhibit further detection of icing if a sufficient insulated barrier is formed. However, the sensor response where no insulating pad is present is strong and can be identified even within the first few seconds of the spray event. Therefore, lower accretion rates than those used in this study should be detectable with this sensor and icing should be detected well before ice has reached levels sufficient to mask the latent heating effect.

This study also confirmed that the MCTCA showed a proper response time as an icing detector. Icing is a phenomenon that occurs over several minutes to hours. Even with a very small amount of spraying, the MCTCA could immediately detect the temperature changed due to the latent heat of fusion for icing accretion. The MCTCA can warn of icing on a specific surface in a very early time without the aid of other sensors.

It is important to note that the convection effects due to ambient wind are ignored in this study. This is mainly because the fluid velocity within the chamber is almost 1 m/s in the test bed. Therefore, Reynold’s number should be small, and the primary heat transfer mechanism is conduction with only a minor convective contribution. Furthermore, the proposed sensor measures the relative temperature differences due to latent heat of fusion during the ice events. Nevertheless, testing within an icing wind tunnel might be needed to investigate how the sensor responds with more convection. It is likely that this increased convection will affect the temperature change detectable by the sensor. However, L. Gao et al. [51] recorded a similar surface temperature change due to latent heat of fusion under more representative operating condition. It reveals that the MCTCA sensor may detect ice on structure surfaces such as wind turbine blades, helicopter blades, and aircraft wings under more realistic environmental conditions. This will be further investigated through measurements performed at a cold climatic wind tunnel in future work.

## 4. Conclusions

In this feasibility study, a novel MCTCA sensor utilising T-type thermoelements was manufactured and tested experimentally to assess its suitability for cold-temperature ice-detection applications and to evaluate it against current industry-adopted approaches. The capability of the sensor in mapping temperatures across a 2D surface was demonstrated. Temperatures measured by the sensor were consistent with those measured using commercially available T-type thermocouples within the ± 0.5 °C error range under the given test conditions. The capability of the sensor for ice-detection was evaluated using an environmental chamber at −15 °C. The considered water droplet characteristics generated by the spray system are approximately 36 μm for MVD and 2.54 g/cm^3^ for LWC at 2 m/s freestream velocity. The considered icing condition is the rime ice condition. Three different cases are considered where water spraying times are different to consider the ice thickness effects. By the series of experimental studies, the following conclusions can be drawn.

(1) MCTCA can detect the surface temperate rise regardless of the surface material which has high (aluminium) and low (wood) thermal conductivities. The temperature measured by the MCTCA for Case II (40 s spraying time) is rapidly increased due to the latent heat of fusion. In order to compare all three test cases, the response of each region is averaged. Temperature increases of (1.0 ± 0.1) °C for plywood board and (0.9 ± 0.1) °C for aluminium plate were detected, respectively, within 20 s after spraying the water droplets.
(2) This study confirmed that the MCTCA can be used as an active sensor. Although this study measured the surface temperature in real time for four hours, the low-power MCTCA can be operated for a much longer time.
(3) The MCTCA can be used as the ice detection system without making any disturbance to the flow field. Although the sensor was embedded in approximately 2 mm thickness epoxy resin, the MCTCA is able to predict the surface temperature increase sufficiently. If the thermal conductivity and the thickness of epoxy resin are correlated, the MCTCA is able to predict the surface temperature more accurately.
(4) The MCTCA displayed a very quick response time as the ice detection system. The MCTCA reliably predicted the temperature increase even though resin was applied and very little ice accumulated. Although the spraying time was varied to 20, 40, and 60 s for this experiment under the specified rime ice condition, the MCTCA detected immediate temperature changes due to the latent heat of fusion. The response time of the MCTCA is much faster than currently employed approaches in industry, e.g., power curve monitoring for wind turbine application. This gives the sensor a huge advantage in applications where detection of ice formation is extremely time-sensitive.

This method of detection is particularly robust compared to other methods, because it directly measures icing in the area of concern rather than at a nearby location which may experience different conditions. There is a low rate of false-positives due to the nature of the latent heat signature, which is only seen when ice forms and is easily distinguished from any other heat inputs. The authors highlight aviation and wind energy generation as two industries which may benefit from this technology. The proposed MCTCA sensor can be embedded on the aerofoil surface of an aircraft wing or a wind turbine blade to provide real-time predictions of temperature distribution and ice accretion. Furthermore, the proposed sensor technology might be applied to any structures operating at cold climatic areas, as this sensor measures only relative temperature changes due to the latent heat of fusion during icing events.

For future work, more detailed sensitivity studies varying resin thickness will be conducted. A better understanding of how the resin thickness affects the signal in both latency and magnitude will allow for more accurate predictions of surface temperature changes due to latent heat. Additionally, a thin-film type sensor will be developed and tested at a cold climatic wind tunnel. The thin-film process allows for the fabrication of smaller junctions which should improve accuracy by reducing the size of “dissimilar regions”. It also lowers the thermal inertia of the sensor, which should reduce latency.

## Figures and Tables

**Table 1:** Water droplet characteristics generated by the spray bottle.

	MVD (μm)	LWC (g/m^3^)
Case 1 ($V_{\infty }=2\;m/s$ and D=0.35 m)	36	2.54
Case 2 ($V_{\infty }=4\;m/s$ and D=0.35 m)	36	1.72

**Table 2:** (**a**) Icing test cases, (**b**) Division of sensor areas into four distinct regions.

**(a)**				
**Test Case**	**Spraying Duration (*s*)**	**No. of Repeats**		
Case I	20	10		
CaseII	40	10		
CaseIII	60	5		
**(b)**				
**Region**	**R1**	**R2**	**R3**	**R4**
Base material	Wood	Wood	Aluminium	Aluminium
Thermal conductivity (W/m K)	0.12–0.04	0.12–0.04	205.0	205.0
Insulation	None	Yes	None	Yes
SSP’s	Avg. (S1,S2,S3)	Avg. (S4,S5)	Avg. (SA1,SA2,SA3)	Avg. (SA4,SA5)
